# Absence of Viable Tumor Cells in Macroscopically Pigmented Regional Lymph Nodes after Immune Checkpoint Inhibitor Therapy in Duodenal Metastasis of Cutaneous Melanoma: A Case Report

**DOI:** 10.70352/scrj.cr.26-0580

**Published:** 2026-10-27

**Authors:** Ryota Unno, Yoshihiro Shirai, Yoshimasa Nobeyama, Tafu Watatani, Takeshi Gocho, Michinori Matsumoto, Munetoshi Akaoka, Isamu Suzuki, Koichiro Haruki, Kenei Furukawa, Toru Ikegami

**Affiliations:** 1 Division of Hepatobiliary and Pancreatic Surgery, Department of Surgery, The Jikei University School of Medicine, Tokyo, Japan; 2 Department of Dermatology, The Jikei University School of Medicine, Tokyo, Japan; 3 Department of Pathology, The Jikei University School of Medicine, Tokyo, Japan

**Keywords:** malignant melanoma, duodenal metastasis, pancreaticoduodenectomy, immune checkpoint inhibitors, regional lymph nodes, lymph node metastasis

## Abstract

**INTRODUCTION:**

Malignant melanoma frequently metastasizes to the gastrointestinal tract, but duodenal metastasis is uncommon, and pancreaticoduodenectomy (PD) for this indication is rare, particularly in the era of immune checkpoint inhibitors (ICIs). Moreover, the histological consequences of ICI therapy within regional lymph nodes draining gastrointestinal metastasis remain poorly documented.

**CASE PRESENTATION:**

A 67-year-old woman with a history of cutaneous malignant melanoma of the thumb developed metachronous ileocecal and duodenal metastases years after the initial resection, during treatment with ICIs. After ICI therapy, she underwent subtotal stomach-preserving PD for the duodenal metastasis. Although preoperative PET/CT showed no regional nodal uptake, the peripancreatic lymph nodes appeared macroscopically black, mimicking nodal metastasis. Histopathological examination revealed fibrosis with CD68-positive, melanin-laden macrophages and no viable tumor cells; Melan-A was negative, and Berlin blue staining excluded hemosiderin, confirming that the pigment was melanin. No viable tumor cells were identified within the regional lymph nodes. The postoperative course was uneventful.

**CONCLUSIONS:**

Macroscopically pigmented “black nodes” after ICI therapy may not represent viable metastasis, and detailed pathological evaluation is essential to avoid misinterpretation. This case also raises the possibility of an immunological role for tumor-draining lymph nodes during ICI therapy, although the implications for the timing of lymph node dissection remain uncertain.

## Abbreviations

EGD
: esophagogastroduodenoscopy
FDG-PET
: fluorodeoxyglucose PET
GI
: gastrointestinal
ICI
: immune checkpoint inhibitor
PD
: pancreaticoduodenectomy
SSPPD
: subtotal stomach-preserving pancreaticoduodenectomy
SUVmax
: maximum standardized uptake value
tdLN
: tumor-draining lymph node

## INTRODUCTION

Malignant melanoma is one of the most aggressive cutaneous malignancies and has a well-known propensity to metastasize to multiple organs, including the GI tract. Although GI metastases are found in up to 60% of patients with cutaneous melanoma at autopsy, they become symptomatic and are diagnosed antemortem in only 1%–4% of cases.^1)^ Within the GI tract, the small bowel is the most frequently involved site, whereas metastasis to the duodenum is comparatively uncommon. In a past autopsy review, the reported distribution of GI involvement included the small intestine in 58%, colon in 22%, stomach in 20%, and duodenum in 12% of cases.^2)^ Because the clinical presentation is often nonspecific or absent, duodenal metastasis of melanoma frequently evades timely detection.

The therapeutic landscape of metastatic melanoma has changed substantially with the introduction of ICIs, which have markedly improved survival in patients with advanced disease.^3)^ In this context, surgical resection of metastatic lesions has been increasingly reconsidered as part of a multidisciplinary strategy, particularly for patients whose systemic disease is well controlled under immunotherapy.^4)^ PD, a highly invasive procedure, has occasionally been reported for duodenal or pancreatic metastasis of melanoma.^5)^ However, these reports largely predate the modern immunotherapy era. To our knowledge, this is the first report of PD performed for duodenal metastasis of melanoma after ICI therapy.

Recently, tdLNs have attracted attention as a central site for the antitumor immune response elicited by ICIs, serving as a reservoir of stem-like CD8-positive T cells that sustain systemic antitumor immunity.^6)^ Accordingly, the optimal timing and extent of lymph node dissection in the perioperative use of ICIs remain under active debate.^7)^ Nevertheless, the histological consequences of ICI therapy within regional lymph nodes draining a GI metastasis have rarely been reported.

Here, we report a case of duodenal metastasis of cutaneous malignant melanoma treated with PD after ICI therapy. Notably, regional lymph nodes that appeared macroscopically black contained only melanophages and no viable tumor cells. We discuss the diagnostic pitfall of these “black nodes” and the implications of our findings for the timing of lymph node dissection in the ICI era.

## CASE PRESENTATION

A 67-year-old woman with a history of cutaneous malignant melanoma was referred to the department of surgery for a duodenal lesion detected on surveillance endoscopy. The patient had been diagnosed with cutaneous malignant melanoma of the left thumb, classified as Stage IA (pT1N0M0), and had undergone surgical resection (Breslow thickness 0.8 mm, ulceration probably absent). She subsequently received adjuvant therapy with interferon beta for 4 years. She remained recurrence-free for over a decade thereafter. Approximately 12 years after the initial operation, the patient presented with left axillary pain and a 3-cm axillary mass. Melanoma metastasis was suspected based on ultrasonography and ^18^F-FDG PET/CT. Excisional biopsy of the mass demonstrated lymph node metastasis of malignant melanoma. Subsequent completion axillary lymph node dissection revealed no residual tumor (Level. 1 [0/2], Level. 2 [0/5]) in the dissected lymph nodes. Systemic therapy with nivolumab was initiated thereafter, and adjuvant nivolumab (2 mg/kg) was continued. Approximately 14 years after the initial operation, ^18^F-FDG PET/CT demonstrated abnormal uptake in the ileocecal region (SUVmax 20.3), and metastasis to the small intestine was diagnosed. Laparoscopic ileocecal resection was performed. Histopathological examination confirmed metastatic malignant melanoma with no evidence of lymph node metastasis. Immunohistochemistry was positive for SOX10, HMB-45, and Melan-A, and negative for AE1/AE3. Following the ileocecal resection, the patient received combination immunotherapy with ipilimumab (3 mg/kg) and nivolumab (80 mg) for 4 cycles. After the second cycle, treatment was suspended because of eosinophilia and resumed after its resolution; the remaining 2 cycles were subsequently completed. She subsequently received maintenance nivolumab (240 mg/body). The only notable adverse event throughout the course was eosinophilia; mild cytopenia was also observed, which became more evident after resumption of therapy. Approximately 16 years after the initial operation, EGD revealed a 40-mm black duodenal tumor in the descending part of the duodenum. The patient was asymptomatic, and the black mass was identified incidentally on routine surveillance EGD (**Fig. 1A**). Contrast-enhanced CT demonstrated wall thickening of the descending portion of the duodenum with a tumor protruding into the lumen (**Fig. 1B**). ^18^F-FDG PET/CT demonstrated abnormal uptake in the duodenal region (SUVmax 8.31) and showed no evidence of other distant metastases (**Fig. 1C**). The tumor was judged to be a resectable, localized metastasis. The case was discussed at a multidisciplinary tumor board, and surgical resection was indicated given the solitary, resectable nature of the lesion and the favorable systemic disease control under immunotherapy. The indication for resection was discussed at a cancer board meeting attended by specialists in dermatology, GI surgery, medical oncology, and radiology. The last dose of ICI was administered approximately 16 weeks before surgery. The patient underwent SSPPD with peripancreatic lymph node dissection for the duodenal metastatic melanoma. The operative time was 317 minutes, and the estimated blood loss was 50 mL. The specimen exhibited a black neoplasm located in the second portion opposite the papilla of Vater (**Fig. 1D**). Black discoloration was noted in the pancreatic lymph nodes, and lymph node metastasis was macroscopically suspected. Pathologically, the tumor was a black lesion measuring 42 × 40 mm. On the cut surface, a white submucosal lesion was identified separately from the black lesion. Both the black and white lesions comprised proliferating, infiltrating tumor cells with round to irregular, enlarged nuclei, prominent nucleoli and lightly eosinophilic cytoplasm (**Fig. 2A**). Immunohistochemically, the tumor was positive for Melan-A (**Fig. 2B**) and S100 (**Fig. 2C**), consistent with malignant melanoma, and was diagnosed as metastasis from the previously treated lesion. The lymph nodes showed partial fibrous degeneration (**Fig. 2D** and **2E**), and an aggregation of CD68-positive macrophages was observed corresponding to these regions (**Fig. 2F**). Melanin deposition was also noted within the fibrotic areas. Berlin blue staining, performed to distinguish melanin from hemosiderin, revealed no hemosiderin deposition (**Fig. 2G**). Melanin phagocytosis by melanophages was observed. In contrast, Melan-A was negative, and no melanocytes were identified at this time (**Fig. 2H**). These findings suggest that the melanin may have been derived from a previous lymph node metastasis of malignant melanoma, with subsequent fibrosis and no residual viable tumor cells. There were no postoperative complications, and the patient was discharged on POD 14.

**Fig. 1:**
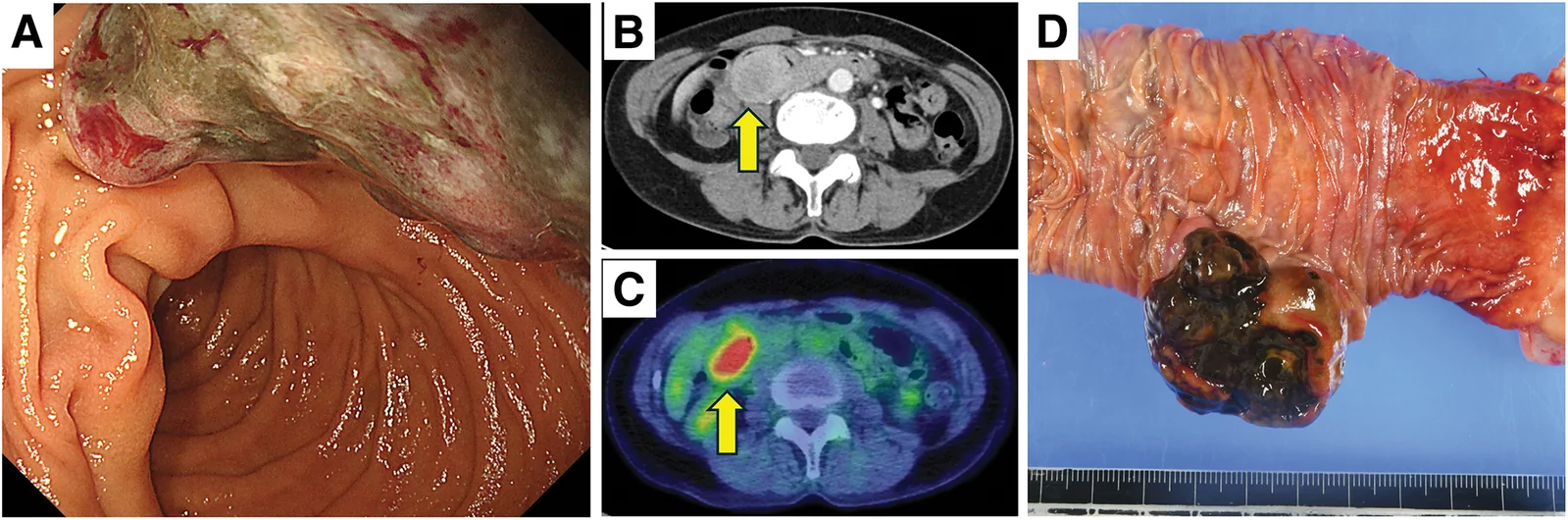
Preoperative imaging of the duodenal metastasis. (**A**) Esophagogastroduodenoscopy shows a black, protruding tumor in the descending part of the duodenum. (**B**) Contrast-enhanced CT demonstrates wall thickening of the descending portion of the duodenum with a tumor protruding into the lumen (arrow). (**C**) ^18^F-fluorodeoxyglucose PET/CT shows abnormal uptake in the duodenal lesion (arrow; maximum standardized uptake value 8.31), with no evidence of other distant metastases. (**D**) The metastatic tumor is located in the second portion of the duodenum.

**Fig. 2:**
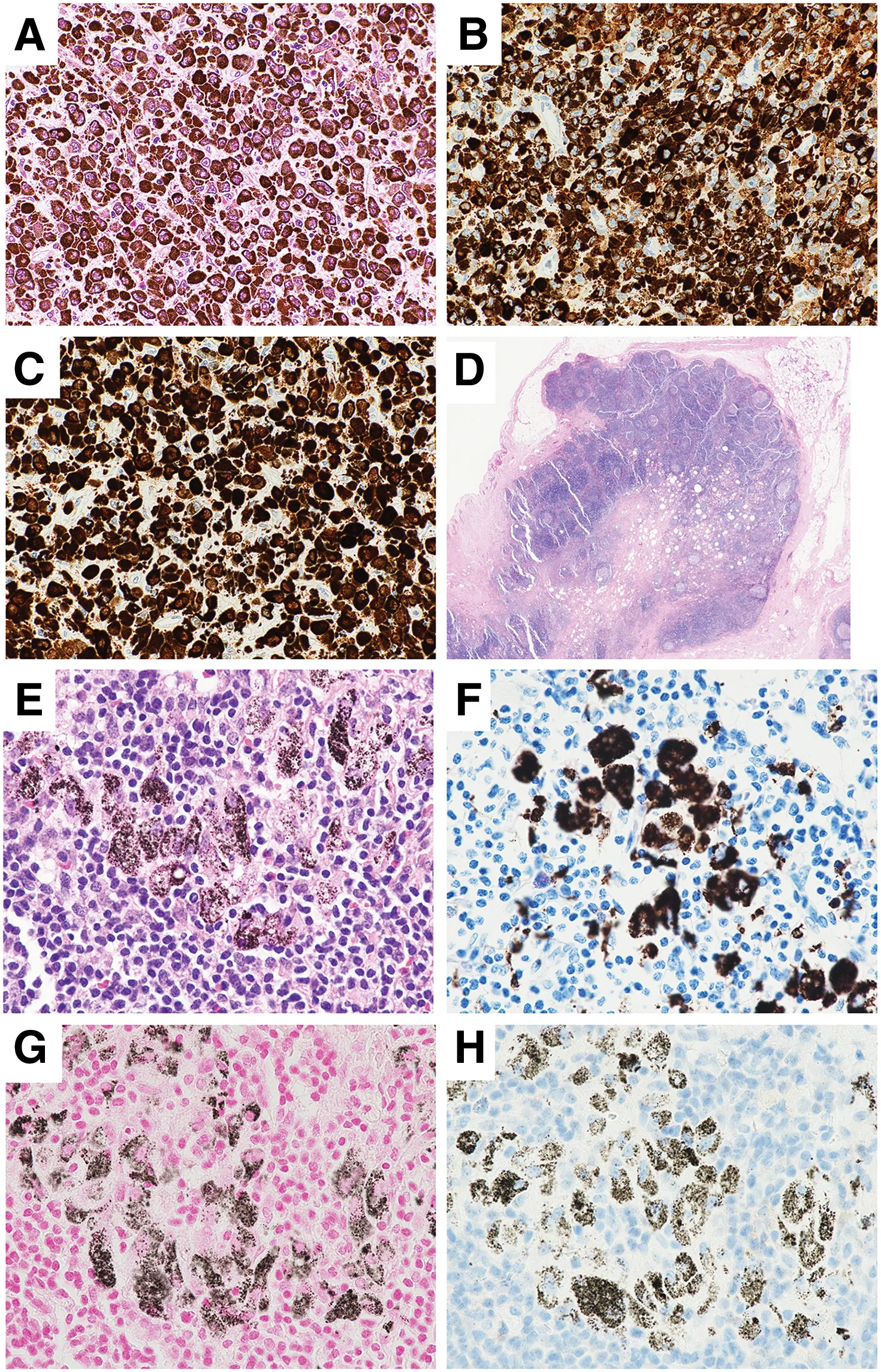
Histopathological findings of the resected specimen. (**A**) Hematoxylin and eosin staining of the duodenal tumor shows proliferating, infiltrating tumor cells with round to irregular, enlarged nuclei and lightly eosinophilic cytoplasm. (**B**) Immunohistochemistry of the duodenal tumor is positive for Melan-A and (**C**) S100, consistent with malignant melanoma. (**D**, **E**) Hematoxylin and eosin staining of the regional lymph nodes shows partial fibrous degeneration with melanin deposition. (**F**) An aggregation of CD68-positive macrophages (melanophages) is observed. (**G**) Berlin blue staining is negative, excluding hemosiderin and confirming that the pigment is melanin. (**H**) Melan-A is negative within the lymph node, and no viable melanoma cells are identified.

## DISCUSSION

In this report, we present a case of duodenal metastasis of cutaneous malignant melanoma in which regional lymph nodes that appeared macroscopically black were found to have no viable tumor cells after ICI therapy. Preoperative ^18^F-FDG PET/CT showed no abnormal uptake in the regional lymph nodes, suggesting the absence of viable nodal metastasis. Intraoperative evaluation revealed a striking black discoloration in the lymph nodes adjacent to the pancreas. Macroscopically, this discoloration resembled that of metastatic nodules. A subsequent histopathological examination revealed fibrous degeneration with aggregates of CD68-positive, melanin-laden macrophages and no viable tumor cells. Melan-A was negative, and Berlin blue staining excluded hemosiderin. As the patient had no history of other pigmented dermatological conditions or tattoos, the most likely explanation is that the melanin deposition originated from a previous nodal metastasis. These findings indicate that no viable tumor cells were identified within the macroscopically pigmented regional lymph nodes. In contrast, the duodenal lesion itself contained viable melanoma cells. Thus, the absence of a viable tumor was limited to the lymph nodes and did not represent an overall pathological response in this patient. To the best of our knowledge, this is the first report to document the pathological evaluation, after ICI therapy, of macroscopically pigmented lymph nodes draining a duodenal metastasis of cutaneous melanoma in which no viable tumor cells were identified. However, these pathological findings do not definitively establish that viable metastatic melanoma had previously existed within these lymph nodes. Alternative explanations should be considered, including passive lymphatic transport of melanin released from the metastatic focus, and regression of microscopic nodal disease that had not been pathologically documented before treatment. Our conclusions are therefore based on the combination of the patient’s history of nodal metastasis and the observed pathological findings and should be regarded as an interpretation rather than a proven mechanism.

This discrepancy between macroscopic and pathological findings may also be related to the immunological role of tdLNs during ICI therapy. Recent studies have shown that tdLNs may function not merely as a route of metastasis, but also as sites for tumor antigen presentation, T-cell priming, and T-cell reactivation following ICI administration.^6,8)^ These findings provide a theoretical basis for administering preoperative immunotherapy in patients with lymph node-positive malignant melanoma. Furthermore, the PRADO trial demonstrated the potential to omit therapeutic lymph node dissection in cases of pathological major response.^9)^

In the present case, the pathological findings in the regional lymph nodes may be consistent with an ICI-induced antitumor response within the tdLN, although this interpretation remains speculative. If tdLNs play a central role in the efficacy of ICIs, the optimal timing and extent of lymph node dissection must be carefully considered. In cases with positive lymph node metastases, adequately exposing metastatic lymph nodes to ICIs prior to lymph node dissection may promote the formation and maintenance of a pool of memory T cells derived from tdLN, which could be beneficial for long-term antitumor immunity. On the other hand, there are reports suggesting that even after lymph node dissection, distant lymph nodes may compensate for the immune response.^10)^ Therefore, at this time, omitting lymph node dissection should be considered only after careful case selection. These considerations regarding the timing and extent of lymph node dissection are hypothesis-generating and cannot be established from a single case; they warrant further investigation in future studies.

Although melanoma has historically been regarded as a systemic disease for which the benefit of metastasectomy was uncertain, the advent of ICIs has renewed interest in resecting metastatic lesions in patients whose systemic disease is well controlled.^4)^ A recent report described microsatellite instability-high duodenal adenocarcinoma treated with ICI therapy, in which a resected specimen showed a pathological complete response, underscoring that ICIs can induce marked pathological regression in GI tumors.^11)^ Because PD is a highly invasive GI surgery, the indication for metastatic lesions must be carefully evaluated. In the present case, PD was considered justified because the duodenal lesion was solitary and resectable, and systemic disease was controlled under immunotherapy. Although new metastatic lesions had emerged during ICI therapy, immunotherapy was continued rather than discontinued. This was because the systemic disease was largely controlled and the emerging lesions were localized and resectable. Therefore, local control by surgery combined with continuation of ICI was considered reasonable. In addition, resumption of ICI therapy after surgery may be considered to maintain systemic disease control.

This report has several limitations. First, regional lymph node metastasis was not histologically confirmed before treatment; therefore, the absence of viable tumor cells should be interpreted as a descriptive observation rather than as definitive evidence of nodal regression, and its immunological interpretation remains hypothesis-generating. Second, the findings are derived from a GI metastasis draining to peripancreatic nodes, an anatomical and immunological context that differs from the cutaneous and nodal basins examined in most prior studies of tdLN biology.

## CONCLUSIONS

In conclusion, we presented a case of duodenal metastasis of cutaneous malignant melanoma that was treated with PD following ICI therapy. The regional lymph nodes, which were suspected to harbor metastasis on visual inspection, showed no viable melanoma cells and may have reflected a response to ICI therapy.

## DECLARATIONS

### Availability of data and materials

All data generated or analyzed during this case report are included in this published article. Further details are available from the corresponding author upon reasonable request.
